# 2-Cyclo­hexyl­idene-*N*-methyl­hydrazine­carbothio­amide

**DOI:** 10.1107/S1600536812042018

**Published:** 2012-10-13

**Authors:** Shahedeh Tayamon, Nurul Ain Mazlan, Thahira Begum S. A. Ravoof, Mohamed Ibrahim Mohamed Tahir, Karen A Crouse

**Affiliations:** a Department of Chemistry, Faculty of Science, Universiti Putra Malaysia, 43400 UPM Serdang, Selangor, Malaysia.

## Abstract

The title compound C_8_H_15_N_3_S has two mol­ecules in the asymmetric unit in which c*is–trans* isomerism is exhibited around the N(NH)C=S bonds. The cyclo­hexyl rings in both mol­ecules adopt a chair conformation. In the crystal, N—H⋯S hydrogen bonding produces dimers, which are inter­connected through further N—H⋯S hydrogen bonds, forming chains along the *b*-axis direction.

## Related literature

For background to the coordination chemistry of dithio­carbazate derivatives, see: Zhang *et al.* (2011 ▶); Khoo *et al.* (2005 ▶); Ravoof *et al.* (2010 ▶). For the synthesis and methodology, see: Tian *et al. (*1997); Tarafder *et al.* (2000 ▶); Tan *et al.* (2012 ▶). For related structures, see: Paulus *et al.* (2011 ▶); Tayamon *et al.* (2012 ▶). For packing arrangements in other cyclohexyl compounds, see: Rohr *et al.* (2009 ▶). For riding constrints, see: Cooper *et al.* (2010 ▶). For charge delocalization, see: Sanderson (1967 ▶). For the synthesis, see: Tian *et al.* (1997 ▶).
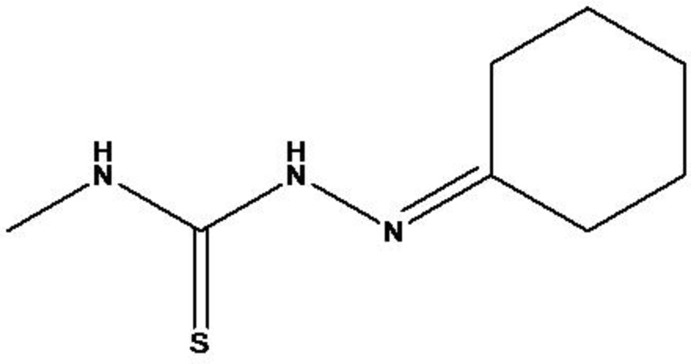


## Experimental

### 

#### Crystal data

C_8_H_15_N_3_S
*M* *_r_* = 185.29
Monoclinic, 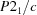

*a* = 10.0538 (3) Å
*b* = 11.0108 (3) Å
*c* = 17.9484 (5) Å
β = 102.132 (3)°
*V* = 1942.52 (10) Å^3^
*Z* = 8
Cu *K*α radiation
μ = 2.56 mm^−1^
*T* = 100 K
0.27 × 0.22 × 0.10 mm

#### Data collection

Agilent Gemini diffractometer
Absorption correction: multi-scan (*CrysAlis PRO*; Agilent, 2011 ▶) *T*_min_ = 0.58, *T*_max_ = 0.77
13859 measured reflections
3754 independent reflections
3414 reflections with *I* > 2.0σ(*I*)
*R*_int_ = 0.025

#### Refinement

*R*[*F* ^2^ > 2σ(*F* ^2^)] = 0.035
*wR*(*F* ^2^) = 0.090
*S* = 0.98
3740 reflections
217 parameters
H-atom parameters constrained
Δρ_max_ = 0.42 e Å^−3^
Δρ_min_ = −0.21 e Å^−3^

### 

Data collection: *CrysAlis PRO* (Agilent, 2011 ▶); cell refinement: *CrysAlis PRO*; data reduction: *CrysAlis PRO*; program(s) used to solve structure: *SIR92* (Altomare *et al.*, 1994 ▶); program(s) used to refine structure: *CRYSTALS* (Betteridge *et al.*, 2003 ▶); molecular graphics: *CAMERON* (Watkin *et al.*, 1996 ▶); software used to prepare material for publication: *CRYSTALS*.

## Supplementary Material

Crystal structure: contains datablock(s) global, I. DOI: 10.1107/S1600536812042018/bg2476sup1.cif

Structure factors: contains datablock(s) I. DOI: 10.1107/S1600536812042018/bg2476Isup2.hkl

Additional supplementary materials:  crystallographic information; 3D view; checkCIF report

## Figures and Tables

**Table 1:** Hydrogen-bond geometry (Å, °)

*D*—H⋯*A*	*D*—H	H⋯*A*	*D*⋯*A*	*D*—H⋯*A*
N205—H2051⋯S101^i^	0.89	2.57	3.4559 (13)	175
N105—H1051⋯S201^ii^	0.87	2.59	3.4484 (13)	169
N203—H2031⋯S201^ii^	0.87	2.76	3.4691 (13)	139

Symmetry codes: (i) 
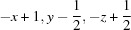
; (ii) 
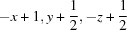
.

**Fig. 1.**
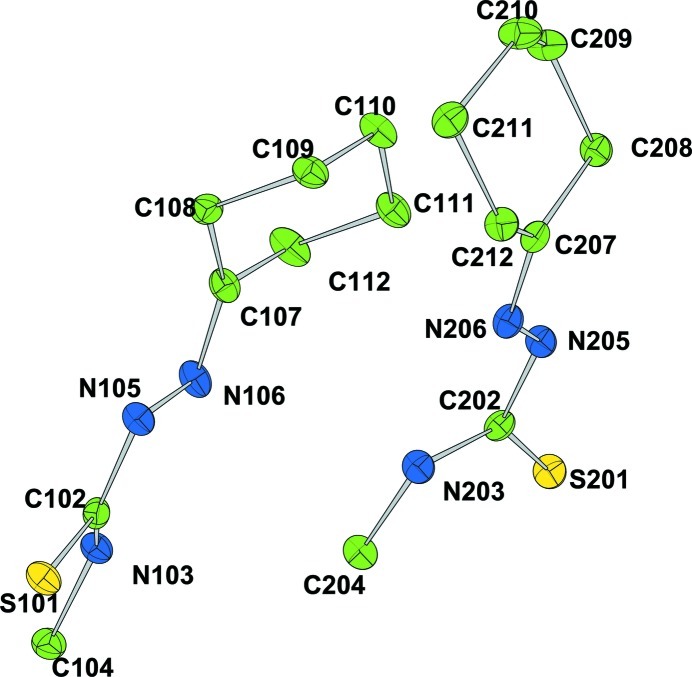
The title compound with displacement ellipsoids drawn at the 50% probability level.

## supplementary crystallographic information

### Comment

To initiate comparative studies between hydrazine carbothioamide Schiff bases (Zhang *et al.*, 2011) and hydrazine carbodithioate derivatives synthesized in our laboratory in our on-going investigations (Khoo *et al.*, 2005; Ravoof *et al.*, 2010, Tan *et al.* 2012, Paulus *et al.* 2011, Tayamon *et al.* 2012), the title compound (C_8_H_15_N_3_S) was synthesized and crystallographically characterized. The compound crystallizes in the monoclinic system, space group *P* 2_1_/c. There are two independent molecules in the asymmetric unit (Fig. 1), in the thione form with C=S bond distances ranging from 1.6953 (15) Å to 1.6982 (15) Å. The values are intermediate between a C—S single bond (~1.82 Å) and a C=S double bond(~1.56 Å) due to charge delocalization (Sanderson, 1967). The C—N and C=N bond distances range from 1.3269 (19) to 1.3596 (19) Å and 1.281 (2) to 1.2818 (19) Å respectively. N—N bond distances vary from 1.3909 (17) to 1.3989 (17) Å, shorter than a single bond and indicating significant π delocalization along the NNC(S)N moiety.

*Cis*-*trans* isomerism is exhibited in the Schiff base around the N(NH)C=S bonds. In both molecules, the methyl group is *cis* to the thione sulfur along Cn02 – Nn03 (n: 1, 2), and the cyclohexyl group is *trans* to the thione sulfur along Cn02 – Nn05. Both cyclohexyl rings are in a chair conformation. The two molecules are twisted relative to one another, as shown by the angle between the planes defined by C108–C109–C111–C112 (largest deviation 0.000 Å) and C208–C209–C211–C212 (largest deviation 0.020 Å) in the respective cyclohexyl ring (83.47°), and S101–C102–N103–C104 (largest deviation 0.009 Å) and S201–C202–N203–C204 (largest deviation 0.013 Å) with a dihedral angle of 27.66°. Molecular packing viewed along the *a* axis shows this orthogonal arrangement of the cyclohexyl rings similar to other subsituted cyclohexyl compounds (Rohr *et al.*, 2009).

The molecular packing is supported by hydrogen bonding through N—H···S interactions (first and second entries in Table 1) creating dimers, which in turn, are also linked through another N—H···S H-bond interaction between dimers (third entry in table 1) creating a chain-like structure along the *b* axis.

### Experimental

The title compound was synthesized following established literature procedures (Tian *et al.*, 1997; Tarafder *et al.*, 2000). 4-methyl-3-thiosemicarbazide (1.05 g, 0.01 mol) dissolved in hot absolute ethanol (30 ml) was added dropwise to an equimolar amount of cyclohexanone (1.04 ml) also in hot absolute ethanol (20 ml). The mixture was stirred for about half an hour at about 340 K and 3 h at room temperature. Pale yellow crystals of the Schiff base suitable for X-ray analysis were obtained after 3 days by keeping the solution at room temperature.

### Refinement

The H atoms were all located in a difference map, but those attached to carbon atoms were repositioned geometrically. The H atoms were initially refined with soft restraints on the bond lengths and angles to regularize their geometry (C—H in the range 0.93–0.98, N—H in the range 0.86–0.89 Å) and *U*_iso_(H) (in the range 1.2–1.5 times *U*_eq_ of the parent atom), after which the positions were refined with riding constraints (Cooper *et al.*, 2010).

### Crystal data

**Table:**

C_8_H_15_N_3_S	*F*(000) = 800
*M**_r_* = 185.29	*D*_x_ = 1.267 Mg m^−^^3^
Monoclinic, *P*2_1_/*c*	Cu *K*α radiation, λ = 1.54180 Å
Hall symbol: -P 2ybc	Cell parameters from 6569 reflections
*a* = 10.0538 (3) Å	θ = 4–71°
*b* = 11.0108 (3) Å	µ = 2.56 mm^−^^1^
*c* = 17.9484 (5) Å	*T* = 100 K
β = 102.132 (3)°	Plate, yellow
*V* = 1942.52 (10) Å^3^	0.27 × 0.22 × 0.10 mm
*Z* = 8	

### Data collection

**Table:**

Agilent Gemini diffractometer	3414 reflections with *I* > 2.0σ(*I*)
Graphite monochromator	*R*_int_ = 0.025
ω scans	θ_max_ = 71.3°, θ_min_ = 4.5°
Absorption correction: multi-scan (*CrysAlis PRO*; Agilent, 2011)	*h* = −12→12
*T*_min_ = 0.58, *T*_max_ = 0.77	*k* = −12→13
13859 measured reflections	*l* = −22→20
3754 independent reflections	

### Refinement

**Table:**

Refinement on *F*^2^	Primary atom site location: structure-invariant direct methods
Least-squares matrix: full	Hydrogen site location: difference Fourier map
*R*[*F*^2^ > 2σ(*F*^2^)] = 0.035	H-atom parameters constrained
*wR*(*F*^2^) = 0.090	Method = Modified Sheldrick *w* = 1/[σ^2^(*F*^2^) + ( 0.05*P*)^2^ + 1.01*P*] , where *P* = (max(*F*_o_^2^,0) + 2*F*_c_^2^)/3
*S* = 0.98	(Δ/σ)_max_ = 0.001
3740 reflections	Δρ_max_ = 0.42 e Å^−^^3^
217 parameters	Δρ_min_ = −0.21 e Å^−^^3^
0 restraints	

### Special details

**Table:**

Experimental. The crystal was placed in the cold stream of an Oxford Cryosystems open-flow nitrogen cryostat (Cosier & Glazer, 1986) with a nominal stability of 0.1 K.Cosier, J. & Glazer, A.*M*., 1986. J. Appl. Cryst. 105–107.

### Fractional atomic coordinates and isotropic or equivalent isotropic displacement parameters (Å^2^)

**Table:**

	*x*	*y*	*z*	*U*_iso_*/*U*_eq_	
S201	0.58434 (4)	−0.01233 (3)	0.30376 (2)	0.0187	
C202	0.60061 (14)	0.13128 (13)	0.27235 (8)	0.0162	
N203	0.53742 (13)	0.22586 (11)	0.29493 (7)	0.0181	
C204	0.44282 (16)	0.21726 (14)	0.34578 (9)	0.0209	
N205	0.67851 (13)	0.15375 (11)	0.22056 (7)	0.0167	
N206	0.70685 (13)	0.27513 (11)	0.20908 (7)	0.0172	
C207	0.76628 (14)	0.30324 (13)	0.15489 (8)	0.0164	
C208	0.80605 (16)	0.22007 (13)	0.09651 (8)	0.0185	
C209	0.76595 (17)	0.27449 (14)	0.01581 (9)	0.0224	
C210	0.81075 (18)	0.40699 (15)	0.01227 (9)	0.0253	
C211	0.75322 (17)	0.48442 (14)	0.06856 (9)	0.0216	
C212	0.80051 (16)	0.43550 (13)	0.14981 (9)	0.0189	
H2042	0.4105	0.2974	0.3532	0.0328*	
H2041	0.4865	0.1830	0.3941	0.0327*	
H2043	0.3665	0.1658	0.3226	0.0325*	
H2082	0.9047	0.2100	0.1102	0.0244*	
H2081	0.7642	0.1400	0.0982	0.0221*	
H2091	0.8084	0.2250	−0.0181	0.0289*	
H2092	0.6676	0.2711	−0.0007	0.0289*	
H2102	0.9105	0.4108	0.0260	0.0327*	
H2101	0.7794	0.4379	−0.0396	0.0332*	
H2111	0.7819	0.5687	0.0665	0.0267*	
H2112	0.6522	0.4812	0.0542	0.0274*	
H2121	0.8989	0.4423	0.1649	0.0247*	
H2122	0.7612	0.4819	0.1862	0.0250*	
H2051	0.7311	0.0945	0.2094	0.0237*	
H2031	0.5539	0.2982	0.2789	0.0238*	
S101	0.10077 (4)	0.43846 (3)	0.32431 (2)	0.0195	
C102	0.09543 (14)	0.30514 (14)	0.27653 (8)	0.0162	
N103	0.03196 (13)	0.20656 (11)	0.29422 (7)	0.0171	
C104	−0.04378 (16)	0.20264 (14)	0.35483 (9)	0.0198	
N105	0.15382 (12)	0.29552 (11)	0.21498 (7)	0.0168	
N106	0.16479 (13)	0.17797 (11)	0.18731 (7)	0.0191	
C107	0.20173 (15)	0.16549 (14)	0.12364 (9)	0.0180	
C108	0.23688 (15)	0.26264 (14)	0.07247 (8)	0.0188	
C109	0.38405 (16)	0.24444 (14)	0.06287 (9)	0.0201	
C110	0.40502 (17)	0.11593 (14)	0.03518 (9)	0.0228	
C111	0.36605 (16)	0.01991 (14)	0.08823 (9)	0.0206	
C112	0.21911 (17)	0.03771 (15)	0.09779 (10)	0.0240	
H1041	−0.0770	0.1213	0.3570	0.0331*	
H1042	0.0137	0.2223	0.4028	0.0329*	
H1043	−0.1208	0.2590	0.3436	0.0325*	
H1081	0.2226	0.3447	0.0915	0.0246*	
H1082	0.1745	0.2523	0.0229	0.0255*	
H1092	0.4454	0.2578	0.1125	0.0256*	
H1091	0.4047	0.3045	0.0275	0.0262*	
H1101	0.5017	0.1051	0.0335	0.0290*	
H1102	0.3480	0.1049	−0.0165	0.0286*	
H1112	0.4287	0.0259	0.1391	0.0258*	
H1111	0.3763	−0.0617	0.0669	0.0248*	
H1121	0.1964	−0.0195	0.1338	0.0317*	
H1122	0.1553	0.0268	0.0483	0.0307*	
H1031	0.0352	0.1396	0.2682	0.0230*	
H1051	0.2103	0.3519	0.2083	0.0243*	

### Atomic displacement parameters (Å^2^)

**Table:**

	*U*^11^	*U*^22^	*U*^33^	*U*^12^	*U*^13^	*U*^23^
S201	0.0218 (2)	0.01267 (19)	0.0224 (2)	−0.00031 (13)	0.00684 (15)	0.00329 (13)
C202	0.0161 (7)	0.0153 (7)	0.0155 (7)	−0.0016 (5)	−0.0003 (5)	0.0006 (5)
N203	0.0214 (6)	0.0127 (6)	0.0214 (6)	0.0004 (5)	0.0069 (5)	0.0022 (5)
C204	0.0213 (8)	0.0199 (8)	0.0225 (8)	0.0000 (6)	0.0072 (6)	−0.0010 (6)
N205	0.0202 (6)	0.0112 (6)	0.0194 (6)	0.0009 (5)	0.0060 (5)	0.0014 (5)
N206	0.0187 (6)	0.0120 (6)	0.0200 (6)	0.0007 (5)	0.0020 (5)	0.0015 (5)
C207	0.0161 (7)	0.0150 (7)	0.0168 (7)	0.0006 (5)	0.0008 (5)	0.0020 (5)
C208	0.0216 (7)	0.0138 (7)	0.0209 (8)	0.0012 (6)	0.0060 (6)	0.0015 (6)
C209	0.0308 (8)	0.0195 (8)	0.0174 (7)	−0.0022 (6)	0.0059 (6)	−0.0001 (6)
C210	0.0362 (9)	0.0208 (8)	0.0199 (8)	−0.0026 (7)	0.0084 (7)	0.0046 (6)
C211	0.0257 (8)	0.0155 (7)	0.0234 (8)	−0.0004 (6)	0.0047 (6)	0.0043 (6)
C212	0.0218 (7)	0.0143 (7)	0.0205 (8)	−0.0010 (6)	0.0045 (6)	0.0004 (6)
S101	0.0230 (2)	0.0150 (2)	0.0221 (2)	−0.00106 (13)	0.00819 (15)	−0.00470 (13)
C102	0.0149 (7)	0.0158 (7)	0.0173 (7)	0.0027 (5)	0.0020 (5)	0.0003 (5)
N103	0.0195 (6)	0.0141 (6)	0.0189 (6)	−0.0002 (5)	0.0069 (5)	−0.0014 (5)
C104	0.0210 (7)	0.0208 (8)	0.0191 (7)	−0.0006 (6)	0.0072 (6)	0.0013 (6)
N105	0.0189 (6)	0.0120 (6)	0.0209 (6)	−0.0016 (5)	0.0070 (5)	−0.0016 (5)
N106	0.0196 (6)	0.0132 (6)	0.0264 (7)	−0.0010 (5)	0.0091 (5)	−0.0030 (5)
C107	0.0149 (7)	0.0176 (8)	0.0223 (7)	−0.0020 (6)	0.0055 (6)	−0.0031 (6)
C108	0.0222 (8)	0.0175 (7)	0.0162 (7)	0.0024 (6)	0.0032 (6)	−0.0008 (6)
C109	0.0232 (8)	0.0181 (8)	0.0211 (7)	−0.0016 (6)	0.0096 (6)	0.0007 (6)
C110	0.0249 (8)	0.0210 (8)	0.0255 (8)	−0.0007 (6)	0.0123 (6)	−0.0037 (6)
C111	0.0240 (8)	0.0150 (8)	0.0249 (8)	0.0004 (6)	0.0094 (6)	−0.0044 (6)
C112	0.0269 (8)	0.0176 (8)	0.0314 (9)	−0.0059 (6)	0.0152 (7)	−0.0069 (6)

### Geometric parameters (Å, º)

**Table:**

S201—C202	1.6982 (15)	S101—C102	1.6953 (15)
C202—N203	1.3269 (19)	C102—N103	1.331 (2)
C202—N205	1.3582 (19)	C102—N105	1.3596 (19)
N203—C204	1.4531 (19)	N103—C104	1.4537 (18)
N203—H2031	0.874	N103—H1031	0.877
C204—H2042	0.959	C104—H1041	0.959
C204—H2041	0.962	C104—H1042	0.955
C204—H2043	0.974	C104—H1043	0.980
N205—N206	1.3909 (17)	N105—N106	1.3989 (17)
N205—H2051	0.889	N105—H1051	0.866
N206—C207	1.2818 (19)	N106—C107	1.281 (2)
C207—C208	1.508 (2)	C107—C108	1.500 (2)
C207—C212	1.504 (2)	C107—C112	1.503 (2)
C208—C209	1.541 (2)	C108—C109	1.538 (2)
C208—H2082	0.977	C108—H1081	0.987
C208—H2081	0.980	C108—H1082	0.982
C209—C210	1.532 (2)	C109—C110	1.529 (2)
C209—H2091	0.979	C109—H1092	0.982
C209—H2092	0.971	C109—H1091	0.969
C210—C211	1.526 (2)	C110—C111	1.528 (2)
C210—H2102	0.982	C110—H1101	0.986
C210—H2101	0.980	C110—H1102	0.990
C211—C212	1.534 (2)	C111—C112	1.535 (2)
C211—H2111	0.975	C111—H1112	0.997
C211—H2112	0.995	C111—H1111	0.990
C212—H2121	0.972	C112—H1121	0.964
C212—H2122	0.975	C112—H1122	0.987
			
S201—C202—N203	122.94 (11)	S101—C102—N103	123.45 (11)
S201—C202—N205	120.43 (11)	S101—C102—N105	120.35 (11)
N203—C202—N205	116.62 (13)	N103—C102—N105	116.16 (13)
C202—N203—C204	124.01 (13)	C102—N103—C104	123.75 (13)
C202—N203—H2031	118.6	C102—N103—H1031	119.0
C204—N203—H2031	117.4	C104—N103—H1031	117.2
N203—C204—H2042	108.2	N103—C104—H1041	107.4
N203—C204—H2041	110.8	N103—C104—H1042	110.7
H2042—C204—H2041	109.9	H1041—C104—H1042	109.0
N203—C204—H2043	109.3	N103—C104—H1043	110.0
H2042—C204—H2043	109.5	H1041—C104—H1043	109.4
H2041—C204—H2043	109.1	H1042—C104—H1043	110.2
C202—N205—N206	116.23 (12)	C102—N105—N106	116.12 (12)
C202—N205—H2051	118.5	C102—N105—H1051	117.6
N206—N205—H2051	121.5	N106—N105—H1051	120.8
N205—N206—C207	119.15 (12)	N105—N106—C107	118.37 (13)
N206—C207—C208	127.94 (13)	N106—C107—C108	128.28 (14)
N206—C207—C212	115.41 (13)	N106—C107—C112	116.74 (14)
C208—C207—C212	116.66 (13)	C108—C107—C112	114.90 (13)
C207—C208—C209	111.22 (12)	C107—C108—C109	109.37 (12)
C207—C208—H2082	107.3	C107—C108—H1081	111.7
C209—C208—H2082	109.4	C109—C108—H1081	111.8
C207—C208—H2081	110.2	C107—C108—H1082	106.5
C209—C208—H2081	110.5	C109—C108—H1082	109.2
H2082—C208—H2081	108.1	H1081—C108—H1082	108.0
C208—C209—C210	112.84 (13)	C108—C109—C110	111.01 (13)
C208—C209—H2091	107.8	C108—C109—H1092	108.3
C210—C209—H2091	109.5	C110—C109—H1092	109.3
C208—C209—H2092	108.6	C108—C109—H1091	109.1
C210—C209—H2092	108.3	C110—C109—H1091	110.9
H2091—C209—H2092	109.8	H1092—C109—H1091	108.2
C209—C210—C211	110.43 (13)	C109—C110—C111	111.52 (12)
C209—C210—H2102	108.9	C109—C110—H1101	109.0
C211—C210—H2102	108.7	C111—C110—H1101	108.6
C209—C210—H2101	109.3	C109—C110—H1102	109.0
C211—C210—H2101	110.2	C111—C110—H1102	109.0
H2102—C210—H2101	109.3	H1101—C110—H1102	109.7
C210—C211—C212	110.40 (13)	C110—C111—C112	111.11 (13)
C210—C211—H2111	110.5	C110—C111—H1112	109.2
C212—C211—H2111	109.6	C112—C111—H1112	109.1
C210—C211—H2112	108.5	C110—C111—H1111	109.0
C212—C211—H2112	109.2	C112—C111—H1111	109.9
H2111—C211—H2112	108.5	H1112—C111—H1111	108.5
C211—C212—C207	111.64 (12)	C111—C112—C107	109.33 (13)
C211—C212—H2121	109.4	C111—C112—H1121	111.2
C207—C212—H2121	106.9	C107—C112—H1121	110.2
C211—C212—H2122	111.4	C111—C112—H1122	110.0
C207—C212—H2122	109.6	C107—C112—H1122	107.2
H2121—C212—H2122	107.7	H1121—C112—H1122	108.8

### Hydrogen-bond geometry (Å, º)

**Table:**

*D*—H···*A*	*D*—H	H···*A*	*D*···*A*	*D*—H···*A*
N205—H2051···S101^i^	0.89	2.57	3.4559 (13)	175
N105—H1051···S201^ii^	0.87	2.59	3.4484 (13)	169
N203—H2031···S201^ii^	0.87	2.76	3.4691 (13)	139

Symmetry codes: (i) −*x*+1, *y*−1/2, −*z*+1/2; (ii) −*x*+1, *y*+1/2, −*z*+1/2.
